# Class-Switch Recombination (CSR)/Hyper-IgM (HIGM) Syndromes and Phosphoinositide 3-Kinase (PI3K) Defects

**DOI:** 10.3389/fimmu.2018.02172

**Published:** 2018-09-26

**Authors:** Rekha D. Jhamnani, Cristiane J. Nunes-Santos, Jenna Bergerson, Sergio D. Rosenzweig

**Affiliations:** ^1^Allergy and Immunology Fellowship Program, National Institutes of Allergy and Infectious Diseases, National Institutes of Health, Bethesda, MD, United States; ^2^Immunology Service, Department of Laboratory Medicine, National Institutes of Health Clinical Center, National Institutes of Health, Bethesda, MD, United States; ^3^Instituto da Crianca, Faculdade de Medicina, Universidade de São Paulo, São Paulo, Brazil; ^4^Laboratory of Clinical Immunology and Microbiology, National Institutes of Allergy and Infectious Diseases, National Institutes of Health, Bethesda, MD, United States

**Keywords:** class-switch recombination, somatic hypermutation, CD40L/CD40 pathway, NF-kB pathway, mTOR pathway, gain-of-function mutations, PIK3CD, PIK3R1

## Abstract

Antibody production and function represent an essential part of the immune response, particularly in fighting bacterial and viral infections. Multiple immunological phenotypes can result in dysregulation of the immune system humoral compartment, including class-switch recombination (CSR) defects associated with hyper-IgM (HIGM) syndromes. The CSR/HIGM syndromes are defined by the presence of normal or elevated plasma IgM levels in the context of low levels of switched IgG, IgA, and IgE isotypes. Recently described autosomal dominant gain-of-function (GOF) mutations in *PIK3CD* and *PIK3R1* cause combined immunodeficiencies that can also present as CSR/HIGM defects. These defects, their pathophysiology and derived clinical manifestations are described in depth. Previously reported forms of CSR/HIGM syndromes are briefly reviewed and compared to the phosphoinositide 3-kinase (PI3K) pathway defects. Diseases involving the PI3K pathway represent a distinctive subset of CSR/HIGM syndromes, presenting with their own characteristic clinical and laboratory attributes as well as individual therapeutic approaches.

## Introduction

Effective humoral immunity relies on the ability of B cells to recognize a wide variety of antigens and respond properly. A diverse BCR repertoire is established by V(D)J recombination in early stages of B-cell development, prior to antigen encounter. Functional commitment of antibodies takes place at a later stage, allowing responses to be tailored upon antigen recognition in a process called class-switch recombination (CSR) (1, 2).

Defective CSR results in an abnormal humoral pattern known as hyper-IgM syndrome (HIGM), characterized by normal or elevated serum levels of IgM in the context of low levels of switched IgG, IgA, and IgE isotypes. A heterogeneous group of primary immunodeficiencies (PID) underlies HIGM, collectively known as CSR defects (1, 3–8).

Two recently described PID, caused by autosomal dominant gain-of-function (GOF) mutations in *PIK3CD* and *PIK3R1*, inducing hyperactivation of the enzyme phosphoinositide 3-kinase δ (PI3Kδ) (9–12), can present with elevated IgM levels in 58–79% of cases, according to the two largest cohorts published to date (13, 14). This finding suggests that PI3K GOF defects disturb CSR and therefore might be part of CSR/HIGM syndromes. As most of the known biology related to the CSR process comes from studies of PID (3, 7), a deeper look into new defects can be instructive to the field.

In this review, antibody maturation steps as well as previously known forms of CSR/HIGM syndromes are briefly described, with special emphasis on the more recently reported PI3K pathway activating mutations. The pathophysiology and clinical manifestations associated with CSR/HIGM syndrome in PIK3CD and PIK3R1 defects are described and analyzed in more detail.

## Class-switch recombination and somatic hypermutation

B-cell development occurs in the bone marrow. Upon completion, naïve B cells express unique B-cell receptors (BCRs) in the form of membrane-bound IgD and IgM. Naïve B cells circulate throughout the blood and lymphatics to secondary lymphoid organs where activation occurs upon ligation of the BCR with its cognate antigen. Subsequently, and mostly with the help of T cells, two critical steps for antibody maturation occur predominantly inside germinal centers: CSR and somatic hypermutation (SHM) (1–4, 7, 8, 15).

In T-cell-dependent responses, CD40 ligand (CD40L), which is expressed on activated CD4^+^ T cells, including T follicular helper cells (Tfh), binds to CD40, a receptor constitutively expressed on B lymphocytes (and monocytes). The CD40L/CD40 engagement in the germinal center, in the presence of the appropriate cytokine milieu, promotes B cells to undergo proliferation, CSR and SHM, through activation of transcription factors, such as nuclear factor-κB (NF-κB). Although in a less effective way, CSR can also be induced in a T-cell-independent manner, via concurrent engagement of BCR or TACI and Toll-like receptors (TLRs) (1–4, 7, 8, 15, 16).

The CSR process involves genomic recombination of the IgM-defining constant (C)μ region in the Ig locus for a downstream Cα, Cγ, or Cε region, coding for the constant regions of IgA, IgG, or IgE isotypes, respectively. Constant regions are flanked by switch (S) regions and CSR occurs when two S regions upstream of a C region undergo switch-region recombination, excising the preceding C regions from the Ig locus. CSR involves a sequential, multi-step process. First, there is a requirement for transcriptional access to the S regions, which allows for activation-induced cytidine deaminase (AID) to catalyze the conversion of cytidine nucleotides to uracil. Next, uracil removal by uracil N-glycosylase (UNG), which, along with endonucleases and the mismatch repair (MMR) machinery, facilitates the generation of double-stranded DNA breaks. Finally, the removal of the intervening DNA as excision circles and repair of the DNA double-stranded breaks, primarily by non-homologous end joining (NHEJ), complete the main steps involved in CSR. Thus, the CSR machinery preserves the Ig variable (V) locus region, thereby maintaining antigen specificity (1–4, 7, 8, 15).

The SHM process randomly introduces mutations into the Ig V regions, altering the affinity for antigens while maintaining the same Ig C isotype. Uracil residues are introduced by AID and can be further modified by UNG. These transitions and transversions generated at the Ig V region are perpetuated during DNA replication, the MMR process, and error-prone polymerase enzymatic activities. SHM additionally allows for affinity maturation by providing a higher diversity of antibodies from which clones with the highest affinity to foreign antigens can be selected. While all HIGM syndromes are determined by CSR defects, SHM abnormalities are not always involved in this group of diseases (1–4, 7, 8, 15).

## Hyper-IgM syndromes

Deficiencies in CD40L, CD40, AID, UNG, NEMO, IkBα, ATM, and PMS2 represent the previously identified forms of CSR/HIGM syndromes. While the clinical manifestations can be variable, the HIGM phenotype can be divided into those disorders that directly affect the CD40/CD40L pathway (B- and T-cells are impacted), and those like AID, UNG, NEMO, IkBα, ATM, and PMS2 where mainly B cells are affected in the CSR/HIGM defect (3, 5–7). The more relevant and comparative characteristics of these diseases are summarized in Table 1.

**Table 1:** Comparison of Hyper-IgM Syndromes.

	**Inheritance**	**Opportunistic infections**	**Lymphoid hyperplasia**	**Lymphoma**	**Autoimmunity**	**CSR defect**	**SHM defect**
CD40L deficiency	XL	+	–	–	+	+	+
CD40 deficiency	AR	+	–	–	+	+	+
NEMO deficiency	XL	+	–	–	+	+	+/−
IkBα deficiency	AD	+	–	–	+/−	+	–
AID deficiency	AR/AD	–	++	–	+	+	+/−
UNG deficiency	AR	–	+	+/−	+	+	–
ATM syndrome	AR	+/−	–	+	+	+	–
PMS2 deficiency	AR	–	–	+	–	+	–
Undefined upstream	AR	–	+	+	+	+	+
Undefined downstream	AR	–	+	–	+	+	–
PI3KCD (APDS1)	AD	+/−	+	+	+	+	+/−
PI3KR1 (APDS2)	AD	+/−	+	+	+	+	+/−

*NEMO, NF-kB essential modulator; AID, activation-induced cytidine deaminase; UNG, uracil N-glycosylase; ATM, ataxia-telangiectasia mutated; PMS2, post-meiotic segregation increased, S. cerevisae 2; PI3KCD, phosphatidylinositol 3-kinase catalytic subunit delta; PI3KR1, phosphatidylinositol 3-kinase regulatory subunit 1; XL, X-linked; AR, autosomal recessive; AD, autosomal dominant; CSR, class-switched recombination; SHM, somatic hypermutation*.

### CD40L deficiency

The first identified CSR/HIGM syndrome was X-linked CD40L deficiency, which is due to a defect in *CD40L* (17–21). The inability of mutated CD40L protein to bind to CD40 affects CD4 T-cell and B-cell interactions, impairing CSR, SHM, T-cell co-stimulation, and development of memory B cells, resulting in a combined immunodeficiency. Patients with this disorder present in infancy with recurrent sinopulmonary infections, and opportunistic infections, such as *Pneumocystis jirovecii* pneumonia (PJP), or *Cryptosporidium*-induced diarrhea and sclerosing cholangitis, which may predispose patients to tumors of the liver, pancreas or biliary tree [reviewed in (3–8, 22)]. In addition to the characteristic immunoglobulin findings, neutrophils can also be low. Activated CD4^+^ T-cells and platelets from patients with X-linked HIGM usually do not express CD40L on their surface; however, some mutations result in defective protein that may still be expressed, thus detecting protein expression is not always a reliable diagnostic tool to rule out this disease, and CD40 binding capacity should be assessed. Past studies have shown that between 7 and 23% of patients with CD40 ligand deficiency have dysfunctional, although detectable, CD40L expression by various methods of testing (5, 23). A normal number of mature B cells is typically observed, but class-switched memory B cells are usually very low to absent. Of note, upon *in vitro* activation, patients' B cells are able to undergo CSR (3). Class-switched CD27^−^IgA^+^ memory B cells can still be produced, although showing limited proliferation (24) and abnormal SHM (25). Pathologically, lymph nodes from these patients are devoid of germinal centers as CD40 and CD40L interaction is imperative for secondary lymphoid organ maturation (3–8, 22).

### CD40 deficiency

CD40 deficiency is rare and inherited in an autosomal recessive (AR) manner (26). Recent reports have enumerated only 17 patients from 13 unrelated families with CD40 deficiency (26, 27). The clinical and immunological phenotype of impaired CSR and SHM is similar to that seen in CD40L deficiency, with one important difference; B cells from CD40 deficient patients are unable to undergo class switching *in vitro* upon activation with agonists and cytokines as per their intrinsic defect. Most patients lack expression of CD40 on the surface of B cells and monocytes (3–8, 26). Although rare, there are reports of CD40 deficient patients in whom a dysfunctional CD40 protein could still be detected (27, 28).

### AID and UNG deficiencies

In contrast to impairments in CD40L/CD40 signaling, defects in the immunoglobulin isotype switching enzymes AID and UNG seem to be primarily limited to deficiencies in antibody production and are mostly inherited in AR manner (29, 30). The most commonly identified cause of AR-HIGM is due to defects in the *AICDA* gene, which encodes AID, an enzyme needed for CSR and SHM in B cells, as described above. Expression of AID is upregulated in response to CD40 signaling, initiating immunoglobulin isotype switching by deaminating deoxycytosine in the immunoglobulin heavy chain switch regions generating deoxyuracils in both DNA strands (31). A limited number of patients carrying *AICDA* heterozygous null mutations that act in a dominant negative way have been associated with autosomal dominant (AD) forms of CSR/HIGM syndromes that preserve SHM and present with a milder clinical phenotype (32, 33). Interestingly, biallelic mutations located in the C terminal region of *AICDA* that do not exert a dominant negative effect have been described in patients with normal SHM, despite drastically impaired CSR (34). Defects in UNG, which removes the deoxyuracils from DNA and initiates the DNA repair pathway, also cause an AR-CSR/HIGM syndrome, and exhibit a skewed pattern of SHM with a predominance of G:C transitions (30). Very few cases of UNG deficiency have been reported. Both AID and UNG deficiencies present clinically with recurrent sinopulmonary infections, mainly caused by encapsulated bacteria. Opportunistic infections and neutropenia are rarely seen. Lymph node hyperplasia and autoimmunity are prominent findings in these diseases; giant germinal centers are typical in AID deficiency. Patients with AID deficiency have also been reported to exhibit gastrointestinal infections, central nervous system infections and arthritis. In both AID and UNG deficiencies patients exhibit no class-switched memory B lymphocytes (3–8, 30, 31).

### NEMO and IkBα defects

X-linked NF-kB essential modulator (NEMO) disease in males can result in CSR deficiency because of the important role of NF-kB in the signaling pathway downstream of CD40 (reviewed in (35)). Since NF-kB is expressed widely and is involved in multiple cell lineages signaling pathways, including both the innate and adaptive immune systems, manifestations of this condition tend to be more diverse and severe. Clinically, male patients carrying hemizygous hypomorphic mutations in this gene can experience a combination of manifestations, such as ectodermal dysplasia and increased susceptibility to viral and bacterial diseases (mainly *Streptococcus pneumoniae*). Osteopetrosis, lymphedema, and mycobacterial infections can also be seen. Interestingly, only about 15% of patients with defects in NF-kB have a HIGM phenotype. Immunoglobulin levels tend to be more variable in this disease as patients have been shown to demonstrate low levels of IgG, low or elevated IgA levels, and normal or increased levels of IgM. Laboratory findings also reflect a defect in switched memory B cells (35).

Heterozygous GOF mutations in *NFKBIA*, encoding IkBα and located downstream of NEMO in the NF-kB signaling pathway, have also been associated to CSR/HIGM syndrome in more than 40% of the patients reported [reviewed in (36)]. These patients share multiple characteristics with those carrying NEMO mutations (e.g., ectodermal dysplasia; viral, bacterial and mycobacterial infection susceptibility) although also presenting some unique features as dysfunctional α/β T cells, very low proportions of memory T cells, and lack of γ/δ Tcells in some but not all affected individuals (36).

### Ataxia-telangiectasia

Ataxia-telangiectasia is an autosomal recessive disorder caused by changes in the Ataxia-telangiectasia mutated (*ATM*) gene, which encodes an enzyme that contributes to DNA mismatch repair. Clinical symptoms include cerebellar ataxia, oculomotor apraxia, telangiectasias and sinopulmonary infections. Because the *ATM* gene is involved in DNA double-stranded repair, patients are also sensitive to ionizing radiation and susceptible to malignancy. Laboratory manifestations can also be variable with some patients presenting with a HIGM phenotype, but most other patients having only reduced levels of IgG2 and IgA. The SHM process is not affected in these patients (37).

### PMS2 deficiency

Though classically associated with Lynch syndrome, an autosomal dominant disorder caused by germline mutations in DNA mismatch repair genes associated with non-polyposis colorectal cancer, biallelic mutations in *PMS2* (post-meiotic segregation increased, *S. cerevisae* 2) result in recurrent severe infections, café-au-lait spots, and a HIGM humoral phenotype. While CSR is defective as in other CSR/HIGM syndromes, SHM is only mildly affected (38) and memory B cells are variably low or normal.

### Undefined Hyper-IgM syndromes

Patients with genetically undefined CSR/HIGM syndromes were reported by Imai et al., and defined as Hyper-IgM type 4 (39). These patients shared clinical and immunologic characteristics with some of the more classical and above-discussed forms of CSR/HIGM syndromes. With the popularization of unbiased genetic testing, many of these undefined CSR/HIGM syndromes can now be attributed to particular underlying molecular defects.

### Treatment of CSR/HIGM syndromes

Since antibody deficiency manifests in all forms of CSR/HIGM syndromes, treatment with immunoglobulin replacement is needed to reduce the frequency and severity of infections. In some cases, prophylactic antibiotics are also needed (40). However, such therapies do not prevent lymphoproliferation, if present, nor is it known if they can mitigate autoimmunity in AR-CSR/HIGM. Patients with CSR/HIGM syndromes that are combined immunodeficiencies (e.g., X-HIGM, CD40 deficiency, NEMO, and NFKBIA) also require *PJP* prophylaxis with trimethoprim-sulfamethoxazole or pentamidine. Neutropenia in X-linked HIGM can be improved using G-CSF (3–8). Hematopoietic stem cell transplant (HSCT) as a treatment for X-linked HIGM syndrome was recently reviewed in a multi-center, international, retrospective (1964–2013), large cohort study of 176 patients. CD40L deficient patients undergoing HSCT showed no statistical differences in terms of overall survival when compared to those not being transplanted. Progress made in HSCT-related issues in recent years, early transplants and better quality of life among transplant survivors, remain the encouraging aspects about HSCT in CD40L deficiency (41).

## Phosphoinositide 3-kinase (PI3K) defects

Activated phosphoinositide 3-kinase δ syndrome (APDS, also known as PASLI- p110δ-activating mutations causing senescent T cells, lymphadenopathy, and immunodeficiency) is caused by heterozygous GOF mutations in *PIK3CD* (APDS1/PASLI-CD) or *PIK3R1* (APDS2/PASLI-R1) that induce hyperactivation of the enzyme PI3Kδ (9–12, 42).

**Figure 1:**
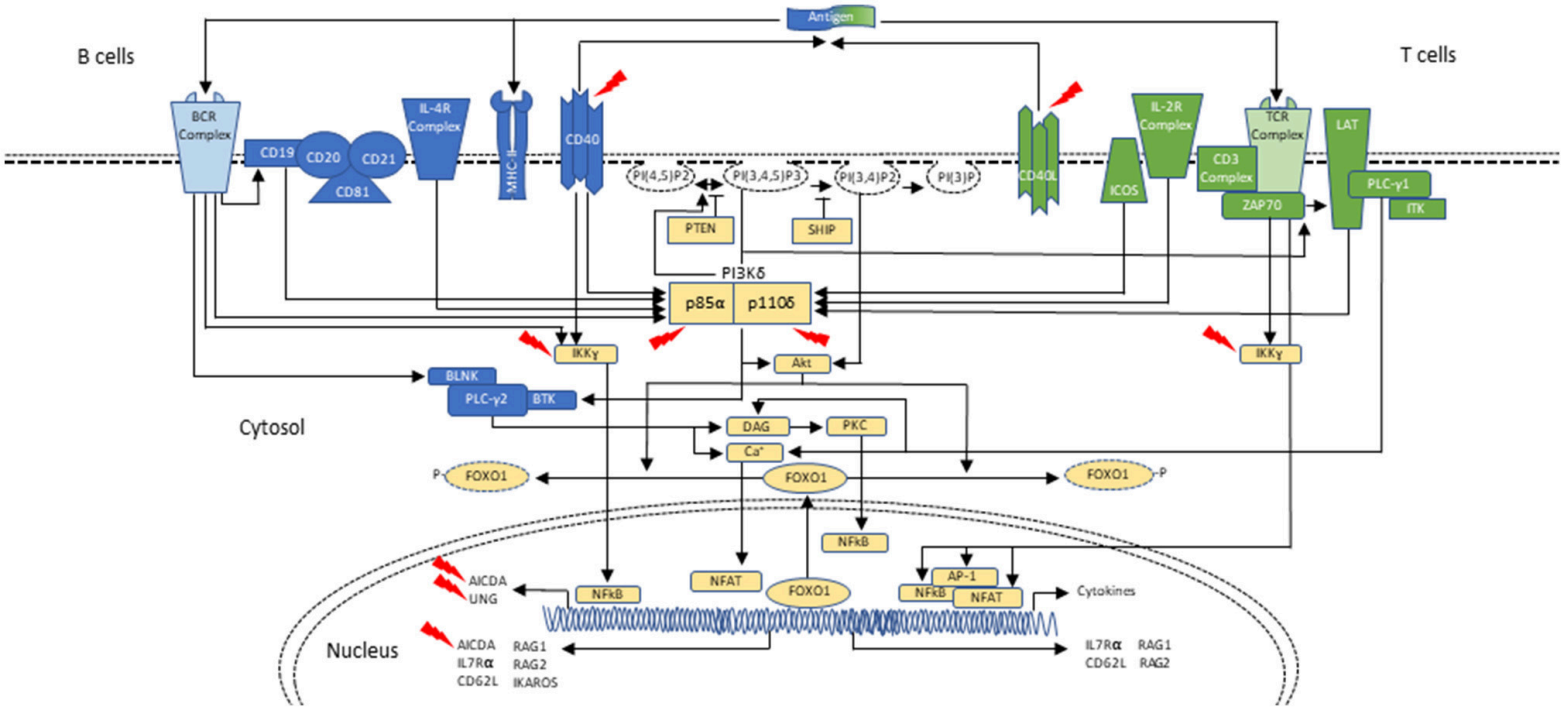
Schematic representation of PI3Kδ activation in B- and T-cells during class-switch recombination. In B cells, PI3Kδ is activated mainly after the BCR (B-cell receptor) cross-linking or upon cytokine stimulation, as with IL-4. CD19 is a co-receptor for the BCR which also p85α can bind. Similar to the T cell, PI3K can signal through Akt to phosphorylate and remove FOXO1 from the nucleus, thus inactivating it. FOXO1 acts as a transcription factor to activate *RAG* genes, *IKAROS, CD62L*, and *AICDA* (critical in CSR and SHM). PI3Kδ signals through mTOR to promote B-cell activation (not shown; reviewed in (42)). In T cells, PI3Kδ is mainly activated upon TCR (T-cell receptor), ICOS and IL-2R stimulation. Akt causes FOXO1 to leave the nucleus and helps with PI3Kδ dependent downregulation of IL-7Rα and CD62L. These changes in turn allow the T cell to exit the lymph node and enter circulation. PI3Kδ via mTOR promotes T-cell activation and T-cell effector phenotypes (not shown). In both B and T cells, PTEN and SHIP act as negative regulators of PI3K (reviewed in (42)). Interaction of T cells and B cells in germinal centers through CD40-ligand (CD40L)/CD40 joining is required for class-switch recombination (CSR) and somatic hypermutation (SHM), two events necessary for antibody maturation. Activated CD4^+^ T cells in the lymph nodes interact with CD40 on B cells, inducing cytokine specific B-cell receptors (IL-R). Signaling through CD40 activates the NF-kB signaling pathway, which ultimately leads to expression of AID and UNG. Mutations affecting components of CD40 mediated B-cell activation can also result in CSR/HIGM syndromes. CD40 has also been associated with PI3K activation. Blue represents signaling elements associated with B cells. Green represents signaling elements associated with T cells. Yellow represents signaling elements that are present in both B- and T- cells. Red arrows point to genes/proteins that when mutated are associated with CSR/HIGM syndromes. Other genes/proteins (e.g., ATM, PMS2, MSH2, and NFKBIA, not included in this figure) can also be associated with CSR/HIGM defects when mutated. BLNK, B-cell linker protein; BTK, Bruton tyrosine kinase; DAG, diacylglycerol; ICOS, inducible T-cell costimulatory; ITK, IL-2 inducible T-cell kinase; LAT, linker for activation of T cells; NFAT, nuclear factor of activated T cells; NF-κB, nuclear factor-κB; PLCγ1, phospholipase Cγ1; PLCγ2, phospholipase Cγ2; PKC, protein kinase C; PTEN, phosphatase and tensin homolog; PI(3)P, phosphoinositide-3-phosphate; PI(3,4)P2, phosphoinositide-3,4-bisphosphate; PI(3,4,5)P3, phosphoinositide-3,4,5-trisphosphate; PI(4,5)P2, phosphoinositide-4,5-bisphosphate; SHIP, SH2 domain-containing inositol-5-phosphatase; ZAP70, zeta-chain-associated protein kinase 70 kD.

Cellular metabolism must be carefully controlled, and in lymphocytes this process requires signaling by a family of enzymes known as phosphoinositide 3-kinases (PI3Ks) that phosphorylate the inositol ring of phosphatidylinositol lipids in the plasma membrane (Figure 1). There are three classes of PI3Ks that have been identified in mammals; most relevant to disorders of the immune system are those involving class IA PI3K enzymes, which consist of a catalytic subunit (p110α, p110β, or p110δ) and a regulatory subunit (p85α, p55α, p50α, p85β, or p55γ). Only p110δ (encoded by *PIK3CD*) is restricted to leukocytes, and it is activated by antigen receptors, co-stimulatory receptors, cytokine receptors and growth factor receptors. Briefly, class IA PI3Ks catalyze the phosphorylation of phosphatidylinositol-(4,5)-bisphosphate (PIP2) to generate phosphatidylinositol-(3,4,5)-triphosphate (PIP3) at the plasma membrane of lymphocytes upon receptor activation and leading to downstream Akt-mTOR signaling (42–45). Phosphatase and tensin homolog (PTEN) and SH2 domain-containing inositol 5′-phosphatase (SHIP) serve as negative regulators of PI3K via dephosphorylation of PIP3 (42, 46, 47) (Figure 1).

The Akt-mTOR pathway is essential for lymphocyte differentiation, function, and maturation. In T cells, PI3Kδ is mainly activated upon TCR (T-cell receptor), ICOS and IL-2R stimulation. Particularly in CD8^+^ T cells, the Akt-mTOR pathway is critical for regulating whether naïve cells differentiate into effector or memory cells by controlling the transition from oxidative phosphorylation, a lower energy yielding process, to aerobic glycolysis, better suited for the rapid growth and proliferation needed by effector T cells. Akt also suppresses the transcription factor FOXO1 function via phosphorylation and through PI3Kδ-dependent downregulation of IL-7Rα and CD62L (42, 47, 48).

PI3Kδ is also highly relevant for B-cell development, antibody responses, and B-cell lymphoma prevention. As seen in T lymphocytes, within B cells, PI3Kδ signaling through phosphatidylinositol lipids and Akt promotes the activation of mTOR and suppresses FOXO1. This transcription factor activates *RAG, IKAROS, CD62L* and *AICDA* genes, which as a group are critical for B-cell development, as well as CSR and SHM (42, 49, 50) (Figure 1). Furthermore, recent studies identifying patients with loss-of-function (LOF) mutations in PTEN, the oppositional counterpart of PI3K, have revealed hyperactivation of the PI3K-Akt pathway with reduced antibody production and T-cell lymphopenia (42, 51).

In APDS, increased PI3K-Akt-mTOR signaling leads to a state of immune dysregulation and immunodeficiency. While the clinical phenotype is varied, most patients present with recurrent sinopulmonary bacterial infections complicated by bronchiectasis, as well as recurrent and severe viral infections from herpes family viruses (9–12, 42). Such clinical manifestations are indicative of both B- and T-cell deficiencies, and as such, APDS is considered a combined immunodeficiency.

The link between APDS and HIGM syndrome was originally reported in one of the two first descriptions of APDS1 (9, 10). In their work, Angulo et al., described a patient clinically diagnosed with HIGM carrying a heterozygous deleterious mutation in *PIK3CD* detected by next generation sequencing. An active search for this particular defect in a cohort of 15 HIGM patients, led to the identification of three more mutated patients (9).

### PIK3CD mutations

Two groups reported the first patients with heterozygous activating mutations in *PI3KCD* as the cause of APDS/PASLI (9, 10). Angulo et al. reported patients found to have a glutamic acid to lysine change at residue 1,021 (E1021K) in p110δ resulting in a GOF mutation with increased lipid kinase activity. The mutation facilitates enhanced phosphorylation of its lipid substrate PIP2, which increases the amount of PIP3, lowering the activation threshold of PI3Kδ. While these patients had recurrent respiratory infections leading to progressive airway damage, they also had herpes viral infections and an increased proportion of effector T cells. Immunophenotyping in this cohort was notable for decreased T and B cells, with increased transitional B cells and decreased class-switched memory B cells, increased IgM levels, low IgG2 levels, and diminished vaccine responses. Furthermore, it was noted that CD4^+^ and CD8^+^ T cells from these patients were prone to cell death, and an increased proportion of T cells had an activated/memory phenotype (9).

Interestingly, the APDS1 patients reported by Lucas et al. was identified from a cohort of individuals with persistent herpes family viremia, lymphoproliferation, and recurrent sinopulmonary infections (10). In this group, three different mutations in *PIK3CD* were found; two novel (N334K and E525K), and the same E1021K mutation reported by Angulo et al. (9, 10). Immunophenotyping was similar to that seen by Angulo et al.; however, normal to high CD8^+^ T-cell counts with progressive CD4^+^ T-cell lymphopenia was observed. Further T-cell phenotyping studies revealed severely reduced naïve and central memory T cells, but increased effector memory T cells and TEMRA (Effector Memory RA^+^) cells. *In vitro*, patients' B cells underwent normal proliferation in response to stimuli, but were unable to secrete class-switched immunoglobulin isotypes (9, 10). This is consistent with the normal to elevated IgM, reduced IgA, and variable IgG levels observed.

Crank et al. found mutations in *PIK3CD* when looking at a small cohort of patients diagnosed with HIGM humoral phenotypes. These patients had high numbers of transitional B cells and plasmablasts. In contrast to previously described CSR/HIGM syndromes, none of the patients found in this cohort experienced opportunistic infections, nor did they have enlarged germinal centers. Additionally, these patients had increased cancer susceptibility with a high prevalence of EBV-negative B-cell lymphomas (52).

**Figure 2:**
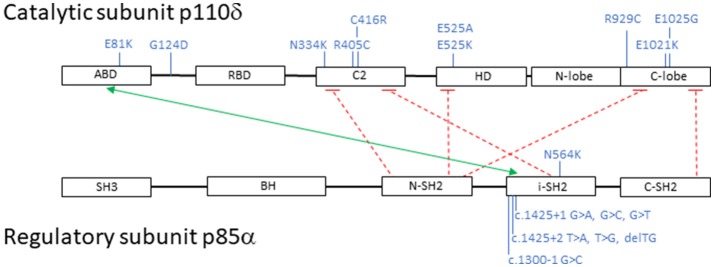
Schematic representation of PI3K catalytic subunit p110δ and regulatory subunit p85α: domains, interactions and mutations. Black boxes represent protein domains: ABD, adaptor-binding domain; RBD, RAS-binding domain; C2, putative membrane-binding domain; HD, helical domain; N-lobe + C-lobe, kinase catalytic domain; SH3, SRC homology 3 domain; BH, breakpoint cluster region homology-domain; N-SH2, N-terminal SRC homology 2 domain; i-SH2, inter-SRC homology 2 domain; C-SH2, C-terminal SRC homology 2 domain. The green arrow points to p110δ and p85α interacting domains; the dashed red lines represent inhibitory contacts between the proteins. In blue, activating mutations affecting p110δ and p85α. Mutations displayed on top of the proteins represent missense changes, mutations displayed below the proteins represent intronic changes.

Subsequently, additional *PIK3CD* mutations have been described and to date 10 heterozygous *PIK3CD* GOF mutations including E1021K, N334K, E525K, C416R, R405C, R929C, E525A, E1025G, E81K, and G124D have been reported. Mutations have now been identified scattered throughout the gene involving not only the kinase domain, but also the helical domain, the C2 domain, the ABD, and the linker region between the ABD and RBD (Figure 2) (11, 14, 42, 51–55). Collectively, and as above mentioned, patients with AD *PIK3CD* GOF defects most commonly present with recurrent respiratory tract infections resulting in bronchiectasis in early childhood, lymphoproliferation, and predisposition to develop B-cell lymphomas. The most commonly reported pathogens are *Haemophilus influenzae, Staphylococcus aureus, Moraxella catarrhalis, Pseudomonas aeruginosa*, and *Klebsiella* species. Recurrent episodes of otitis media are also observed, sometimes resulting in hearing loss. A small percentage of infections consisted of conjunctivitis, orbital cellulitis, skin abscesses, and dental abscesses. Lymphadenopathy, mucosal lymphoid hyperplasia, and hepatosplenomegaly are also common features. A broader range of viral infections including varicella zoster, herpes simplex virus, adenovirus, and *molluscum contagiosum* have been reported. Increased susceptibility to herpes virus family infections in these patients is likely due to the combinatory effect of reduced long-lived memory CD8 T cells and increased terminally differentiated effector CD8 T cells (11). While patients with *PIK3CD* GOF mutations had normal/high EBV-specific CD8 T cells (based on tetramer staining), they were predominantly effector memory terminal cells (CCR7^−^/CD45RA^−^) also showing signs of increased activity (by CD38 staining). Persistent Akt hyperactivation was hypothesized as the driver for the increased CD8 T-cell proliferation, that in turn determined higher terminally differentiated CD8 T-effector cells, increased senescent CD8 T cells and decreased long-lived memory CD8 T cells, altogether resulting in impaired control of EBV- and CMV-infected cells (11). Although rare, opportunistic infections in these patients include *Cryptosporidium parvum* and toxoplasmosis. Autoimmune features included cytopenias, glomerulonephritis, and thyroid disease. Aside from EBV-positive lymphoma, other malignancies included EBV- negative diffuse large B-cell lymphoma, Hodgkin lymphoma, nodal marginal zone lymphoma, lymphoplasmacytic lymphoma, and cutaneous anaplastic large cell lymphoma (14, 42, 56). Increased IgM levels and low IgA, IgG, or IgG2 levels were again a defining laboratory finding in symptomatic patients. Flow cytometry revealed similarities to previously reported immunophenotyping: reduced CD4 T-cell counts, increased effector memory CD8 T-cell counts, and increased transitional B cells that can be linked to abnormal B-cell precursor maturation in the bone marrow (14, 42, 54).

### PIK3R1 mutations

Heterozygous loss-of-function mutations in *PIK3R1*, encoding the p85α regulatory subunit, also result in hyperactivation of PI3Kδ signaling due to loss of regulatory control on the catalytic p110δ subunit. Almost all reported patients have splicing mutations leading to skipping of exon 11. More recently, an additional missense mutation located in the SH2 domain of *PIK3R1*, N564K, was described by Wentink et al. and is predicted to affect binding to p110δ (53). Because the net result of these mutants determine an increased PI3Kδ activity, they are considered GOF mutations (11–13, 42).

Similar to patients with mutations in *PIK3CD*, APDS2/PASLI-R1 patients also present with recurrent bacterial sinopulmonary infections, EBV/CMV viremia, chronic lymphoproliferation, and increased risk of lymphoid malignancy. Elevated IgM levels accompanied by low IgG and IgA are frequent, along with reduced naïve T cells and class-switched memory B cells, increased levels of senescent CD8^+^ T cells and transitional B cells making patients carrying mutations in *PIK3CD* and *PIK3R1* clinical phenocopies (11–13, 42). Consistent with what is seen in *PIK3CD* mutations, patients' lymphocytes revealed hyperactivation of PI3K, Akt, and mTOR. Additionally, increased activation-induced cell death was also seen in APDS2 patients' T cells, with a high proportion of these T cells expressing the senescence marker CD57 (11, 12, 42).

In a cohort study of APDS2, Elkaim et al. reported growth retardation in 14/31 (45%) patients (13). Growth problems are not frequent in APDS1 (14, 57). This particular defect is likely related to non-immunological roles of p85α. In support of this hypothesis, autosomal dominant mutations in the last exons of *PIK3R1* cause SHORT syndrome (short stature, hyperextensibility of joints and/or hernias, ocular depression, Rieger anomaly, and delays of tooth eruption). Other features consistently seen in patients with SHORT syndrome include mild intrauterine growth restriction, characteristic facies, lipodystrophy and insulin resistance in adolescence progressing to diabetes mellitus in early adulthood. These patients do not typically present with a HIGM humoral phenotype. Immunoblot analysis has shown that levels of p85α were ~50% lower in SHORT syndrome patients than in controls (58). Normal PI3K activity is critical for adipose tissue differentiation and insulin signaling, thus explaining the lipodystrophy and abnormal glucose tolerance in these patients (59). Interestingly, three patients presenting concomitant features of SHORT syndrome and APDS2 have been described, two of whom showed a HIGM phenotype (60, 61).

While the non-immunological role that p85a plays is certainly highlighted by those affected with SHORT syndrome, evidence for its importance in lymphocyte function is also clearly shown in a patient with homozygous, biallelic, premature stop codon mutations in *PIK3R1* leading to complete absence of p85α. This patient had colitis and history of *Campylobacter* infection, in the context of absent B cells with resulting agammaglobulinemia. Early in life, erythema nodosum and arthritis were also observed (62).

### Class-switch recombination in PI3K GOF defects

Early insights on the impact of hyperactivation of PI3Kδ in CSR relate to murine models lacking PTEN expression in B cells. These mice showed a HIGM phenotype with defective CSR that was partially dependent on FOXO1 suppression and reduced AID expression. The CSR process was not completely corrected by ectopic AID expression, but was additionally restored in the presence of a specific PI3Kδ inhibitor (63, 64). These findings were further supported by another mouse model in which mature B cells lacking FOXO1 also expressed lower levels of AID and failed to produce class-switched antibodies, which in this setting could not be rescued by PI3Kδ inhibition (65). Considering the suppressive effect of the PI3K/Akt pathway on FOXO1 and the importance of AID in the CSR process, these results suggest that reduced expression of AID due to inactivation of FOXO1 contribute to the CSR impairment seen in APDS. As shown in other murine models, specific ablation of FOXO1 in germinal center B cells results in abnormal CSR despite normal expression of AID. In these models, germinal center dark zones were ablated and even though proliferation and SHM were preserved, affinity maturation was impaired. Thus, in the absence of FOXO1 in germinal center B cells', AID was normally expressed and yet not sufficient to maintain appropriate CSR. These experiments suggest some additional roles of FOXO1 on AID function, and therefore CSR (66, 67).

Avery et al. (68) developed a novel Pi3kcd^E1020K^ GOF mouse model by CRISPR/Cas9-mediated genome editing. Hyperactivation of PI3Kδ in B cells was documented by increased pAkt and pS6. B cells from mutant mice showed defective class switching and Ig secretion both upon *in vitro* (anti-CD40 + IL-4, LPS, or LPS + TGF-β) and T-dependent *in vivo* stimulation. Of note, Pi3kcd^E1020K^ GOF B cells behaved similarly to WT cells regarding proliferation, germinal center formation, SHM and affinity maturation. Induction of *AICDA* mRNA in B cells following stimulation with anti-CD40/IL-4 was significantly reduced in mutants compared to WT and positively correlated with the percentage of IgG1^+^ B cells generated after activation. *In vitro* treatment with the p110δ inhibitor leniolisib (69) completely restored the class-switch defect in Pi3kcd^E1020K^ GOF B cells, as well as *AICDA* expression levels and secretion of IgG and IgA.

In accordance with the highly prevalent HIGM phenotype observed in patient cohorts, most human studies of APDS found reduced class-switched B cells in the periphery and impaired CSR *in vitro* (10, 13, 14, 53, 68). Increased Akt phosphorylation in B cells, even without stimulation, was another consistent finding (9, 10, 53, 70). While Angulo et al. (9) observed normal SHM in two *PI3KCD* GOF patients, as later reported in the corresponding murine model (68), variable frequencies of SHM were found in seven APDS patients evaluated by Wentink et al. (53). Altogether, these data suggest that SHM can show some variability in PI3K mutated patients although it seems more consistent in the mouse model.

APDS patients studied by Avery et al. showed reduced *AICDA* expression, in line with their Pi3kcd GOF murine model, as well as with previous experimental models of increased PI3kδ activity (63, 64, 68). This is in contrast to normal expression levels of *AICDA* reported in *PIK3CD* GOF patients by Lucas et al. (10). Targeting of AID to switch regions was assessed in one APDS cohort and shown to be normal (53).

Specific PI3Kδ inhibition is under investigation in APDS patients (71). In a Phase1-phase 2 leniolisib trial, a progressive decrease in patients' serum IgM levels was observed along with a reduced need for IgG supplementation. These findings might reflect a restoration in CSR, however further studies will be needed to confirm and solidify this effect.

Besides this growing evidence pointing to an intrinsic B-cell defect underlying diminished CSR in APDS, given previous data linking PI3Kδ signaling to Tfh cells generation (72) and the requirement of T- and B-cell interaction for effective CSR (2), it is reasonable to speculate whether T-cell related dysfunction additionally impairs CSR in these defects.

To investigate the hypothesis that activating PI3K defects could lead to anomalous germinal center function, Preite et al. (73) generated another mouse model expressing p110δ^E1020K^. In their animal model increased numbers of Tfh and germinal center B cells were found, along with impaired CSR after immunization. Germinal centers dark zones were poorly formed, with extensive infiltration of Tfh cells. Of particular interest to their model was the observation that, in the setting of hyperactive PI3K/Akt pathway, differentiation to Tfh cells was facilitated by a strong suppression of FOXO1 that occurred independently of ICOS engagement. Adoptive transfer of mutant naïve transgenic T cells into wild type mice resulted in higher Tfh differentiation. These Tfh cells did not lead to increased number of germinal center B cells and were able to provide normal B cell help *in vitro*. Similar findings of disrupted germinal centers infiltrated by Tfh cells were described in APDS1 patients by Coulter et al. (14). Interestingly, while studying an APDS2 patient, Di Fonte et al. found significantly reduced numbers of germinal center Tfh cells in tonsillar tissue (74).

### Activating PI3K defects compared to other CSR/HIGM syndromes

Clinically, all CSR/HIGM syndromes share an increased risk of recurrent bacterial infections, mainly reflecting the absence of isotype specific protective functions (3, 7). Variable impairment of other immune functions that are not a consequence of defective CSR define unique infection patterns observed in specific defects. For instance, the lack of proper interaction between T lymphocytes and monocytes in CD40/CD40L deficiencies confers an increased risk for *Pneumocystis jiroveci* pneumonia, an opportunistic infection not frequent in others CSR/HIGM defects (3). An intact CD40/CD40L interaction is also required to clear *Cryptosporidium parvum* infection of bile duct epithelium leading to sclerosing cholangitis (7). Although sclerosing cholangitis has also been described in a few APDS patients, there was no link to *Cryptosporidium* species infection and therefore is believed to be primary rather than associated to infections. This was hypothesized to be due to the fact that genes associated with primary sclerosing cholangitis often converge at the PI3K/Akt signaling pathway (42, 75). Recent data showed that persistent viral infections (particularly herpesviruses), a hallmark in activating PI3Kδ defects but not common among other CSR/HIGM syndromes, arise from exhaustion of cytotoxic CD8^+^ T cells and NK cells (76, 77).

Increased autoimmune manifestations are seen in almost all CSR/HIGM syndromes including PI3K defects (3, 7, 13, 14, 78). Functional CD40L seems to be necessary for normal peripheral B cell tolerance, whereas AID is important for both central and peripheral B cell checkpoints. Natural IgM antibodies are also found in AID deficient patients (79). Increased numbers of autoreactive B cells were seen in the GOF PI3Kδ mouse model (73).

Concerning the immunological phenotype, some similarities as well as differences can be seen between PI3K defects vs. the other CSR/HIGM syndromes. While a reduction in class-switched B cells is common to all CSR/HIGM defects, reduced total number of circulating B cells, along with increased transitional B cells are distinctive features of PI3K defects (3, 7, 42, 78). Memory B cells (CD27^+^), that are shown to be reduced in PI3K defects (10, 42, 53) and absent or very low in CD40/CD40L deficiencies, are normal in AID/UNG deficiencies (3). While total T-cell numbers are usually normal in CD40/CD40L and AID deficiencies, they appear to be reduced in PI3K defects. PI3K defects also show a unique distribution of T-cell subsets, with skewing toward effector and exhausted phenotypes. An inverted CD4/CD8 ratio is also typical, due to both increased CD8^+^ and reduced CD4^+^ T cell counts (especially in the naïve compartment) (13, 14, 42). Reduced naïve CD4^+^ T-cell counts and inverted CD4/CD8 ratios can also be seen in AID deficiency, although less strikingly than in PI3K defects. Circulating Tfh cells, increased in APDS (14) and AID deficient patients (80), are markedly decreased in CD40L deficient patients (3, 81).

Germinal centers' architecture also helps to distinguish between different CSR/HIGM syndromes. While generally absent in CD40/CD40L deficiencies and giant in AID deficiency (3, 79), they appear to be present but disrupted in APDS patients (14, 82).

### Treatment of PI3K GOF defects

Treatment for APDS patients includes prophylactic measures, such as antibiotics, antivirals, and antifungals, as well as immunoglobulin replacement. Most patients undergo immunosuppressive therapy aiming to control lymphoproliferation and/or autoimmunity. Rapamycin was a frequently used immunossupressor in the ESID-APDS-registry cohort, showing positive results in controlling benign lymphoproliferation, but not as effective for gastrointestinal manifestations and cytopenias (83). Rituximab has been successfully used in the treatment of cytopenias and lymphoproliferation, but persistent B-cell lymphopenia can be a common outcome (14).

Therapeutics created for the treatment of leukemia and lymphoma, such as idelalisib, duvelisib, or ibrutinib block PI3Kδ either directly or indirectly, making them exciting potential options for treating patients with APDS (84). Leniolisib, a PI3Kδ inhibitor has been explored as a therapeutic option in patients with PIK3CD GOF mutations. Six patients were treated over a 12-weeks period and demonstrated decreased lymphadenopathy and splenomegaly. Decreased Akt phosphorylation in affected patients' T cells, with noted reductions in transitional B cells, senescent T cells, and IgM levels were also observed (71). However, it has been shown in both mouse and human B cells that by modulating PI3Kδ activity there is a resulting enhancement of AID expression leading to increased SHM and chromosomal translocation to the IgH locus as well as to other AID off-target sites. Thus, PI3Kδ blockade via an AID-dependent mechanism increases genomic instability. Given that such inhibitors could be administered to patients for extended periods of time, several concerns arise including the potential for secondary oncogenic mutations or translocations, and accelerated resistance to targeted therapy by increasing the mutational rate (84). Furthermore, idelalisib, an oral selective inhibitor of PI3Kδ, has been reported to cause enterocolitis and a rash mimicking graft-versus-host disease (85).

When focused on curative treatment, Nademi et al. reported an 81% survival among 11 APDS patients who underwent HSCT in seven pediatric centers. Acute GvHD was a common complication (81%), 2 patients presented low chimerism, and 2/3 of the surviving patients are off immunosuppressive therapy and immunoglobulin replacement (86).

## Conclusions

Diseases involving the PI3K pathway due to *PIK3CD* and *PIK3R1* GOF mutations, have recently been highlighted as forms of combined immunodeficiency compromising both the T- and B-cell compartments. Common clinical features include recurrent bacterial respiratory tract infections, EBV/CMV viremia, T-cell lymphopenia, memory B-cell deficiency, increased transitional Bcells and elevated IgM levels accompanied by low IgG and IgA levels. Due to elevated IgM levels and low IgG, IgA, and IgE levels, PI3K diseases ultimately fit under the umbrella of the CSR/HIGM syndromes.

While all the diseases presenting with HIGM humoral phenotypes described in this review involve by definition, CSR defects, this mechanism is not univocally disrupted in all these defects. Expression and function of CD40L/CD40 and AID are crucial to this process, and when either of them is altered, they markedly affect its outcome. In PI3K-related defects, evidence points at AID expression and function through FOXO1 regulation playing a central role in CSR integrity. In terms of SHM, this process is generally preserved when defects involve *AD-AICDA, UNG, NEMO, NFKBIA, ATM*, and *PMS2*, but shown to be variably affected in patients carrying PI3K-associated diseases, although more consistently normal in animal models (9, 53, 68). Whereas, deficiencies in the CD40L/CD40 and NF-kB pathways are characterized by opportunistic infections, patients with defects in AID and UNG generally do not experience such complications. Patients with *PIK3CD* and *PIK3R1* mutations appear to be intermediate between the above-mentioned defects in terms of opportunistic infections susceptibility. Over time, as with many other immunodeficiencies, autoimmunity has become a more relevant feature demonstrating overall immune dysregulation within HIGM syndromes in general and PI3K defects in particular.

In conclusion, the CSR/HIGM syndromes encompass a wide variety of diseases, each with their own defining features. Diseases involving the PI3K pathway are indeed combined immunodeficiencies, as well as a subset of CSR/HIGM syndromes, presenting with their own characteristic clinical and laboratory attributes as well as individual therapeutic approaches.

## Author contributions

RDJ wrote the first draft. RDJ, CJN-S, JB and SDR contributed and reviewed all the data presented. JB and SDR supervised the project.

### Conflict of interest statement

The authors declare that the research was conducted in the absence of any commercial or financial relationships that could be construed as a potential conflict of interest.
